# Generation of Human Breg-Like Phenotype with Regulatory Function In Vitro with Bacteria-Derived Oligodeoxynucleotides

**DOI:** 10.3390/ijms19061737

**Published:** 2018-06-12

**Authors:** Jorge Gallego-Valle, Verónica Astrid Pérez-Fernández, Rafael Correa-Rocha, Marjorie Pion

**Affiliations:** Immuno-Regulation Laboratory, University General Hospital Gregorio Marañón, Health Research Institute Gregorio Marañón (IiSGM), Medicine and Experimental Surgery Building, Calle Máiquez, 9, 28009 Madrid, Spain; jorge.elgallego@hotmail.es (J.G.-V.); veronica.astrid@iisgm.com (V.A.P.-F.); rafael.correa@iisgm.com (R.-C.R.)

**Keywords:** Breg-like B cells, IL-10-producing B cells, suppressive function

## Abstract

Regulatory B cells (Bregs) participate in auto-tolerance maintenance and immune homeostasis. Despite their impact on many diseases and due to the difficulty to define them, knowledge about their origin and their physiological inducers is still unclear. The incomplete understanding about the generation of Bregs and their limited numbers in periphery make it difficult to develop Breg-based therapy. Therefore, identifying factors that promote their development would allow their ex-vivo production in order to create new immunotherapy. This project aims to test the capacity of several cytokines (Interleukin 1-beta (IL-1β), Granulocyte Macrophage Colony-Stimulating Factor (GM-CSF), and Cluster of differentiation 40 ligand (CD40L)) and bacteria-derived oligodeoxynucleotides (CpG-ODN), alone or in combination, to generate B cells with regulatory phenotype and function. We have demonstrated that the Breg-associated phenotypes were heterogeneous between one and other stimulation conditions. However, the expression of other markers related to Bregs such as IL-10, CD80, CD86, CD71, Programmed cell death-1 (PD-1), and Programmed death-ligand 1 (PD-L1) was increased when cells were stimulated with CpG alone or in combination. Moreover, stimulated B cells presented a suppressive function on autologous activated peripheral blood mononuclear cells (PBMC) proliferation. Therefore, this work is the first step to demonstrate the feasibility to induce functional Breg-like cells in vitro and will then facilitate the way to produce Breg-like cells as a potential future cellular therapy.

## 1. Introduction

Homeostasis of the immune system must be highly regulated via pro-inflammatory and anti-inflammatory mechanisms in order to keep the body prepared to fight off infection and to maintain a state of unresponsiveness to their own antigens. Continuous inflammatory responses due to injuries or infections lead to tissue and organ damage. Therefore, a rapid anti-inflammatory response is necessary to restore immune homeostasis [1]. During the last decade, multiple regulatory responses, whose actions are focused on limiting hyper-activation and inflammatory reactions, have been described. Until now, regulatory T cells (Treg) are the best described T-cell subset with regulatory function [2,3]. Contrarily to Treg, regulatory B cells (Breg) have been less studied due to difficulties in their determination. Currently, although Treg were defined as Foxp3+ T cells, a specific transcription factor has not been identified for Bregs. However, even if the origin and definition of Bregs are still controversial, their function in maintaining self-tolerance, immune homeostasis, and the prevention of autoimmunity has been clearly identified in different pathologies [4,5,6,7]. In humans, it was demonstrated that different B-cell subsets such as CD19^+^CD24^hi^CD38^hi^ [6,8], CD19^+^CD24^hi^CD27^+^ [9], CD19^+^CD5^+^CD1d^hi^ [10], or CD19^+^CD73^neg^CD25^+^CD71^+^ [11] could exert a regulatory function Thereby, a large variety of regulatory B-cell subsets have been identified, which could lead to some confusion. However, intracellular IL-10 expression can only easily characterise the suppressive B-cell population in mice and humans. More recently, other mechanisms related to the expression of CD80/CD86, indoleamine 2,3-dioxygenase (IDO), or PD-L1 but independently of IL-10, which might be responsible for Breg function have been described [12,13].

Surely, difficulties and diversity in Breg definition could come from their generation and isolation. The origin of the different Breg subsets is subject to debate. Nowadays, the main hypothesis is that Bregs can be generated from any B cell subset regardless of maturity or differentiation status, but only depending on the microenvironment [14]. This theory is supported by several studies that showed that distinct B cells with regulatory function could be induced by different stimuli [15,16,17]. Therefore, due to the potential plasticity of these cells, Breg cells might be generated in vitro, which would be a formidable opportunity to develop a new therapeutic source of Breg by the discovery of their physiological inducers.

In this work, we tested several cytokines and bacteria-derived oligodeoxynucleotides (CpG) to generate B cells with regulatory phenotype and function. Human primary B cells were stimulated with the cytokines IL-1β, CD40L, GM-CSF, and the bacterial-derived factor CpG, alone or in combination. Two of the Breg-associated phenotypes were followed in this work and their suppressive capacity was studied. The two Breg-associated phenotypes were not clearly generated by any of the stimuli used, but other markers related to Bregs such as IL-10, CD80, CD86, CD71, PD-1, and PD-L1 were expressed when cells were stimulated with CpG or with a combination of stimuli containing CpG. In addition, these CpG-stimulated B cells presented a suppressive function on peripheral blood mononuclear cells (PBMC) proliferation in an autologous manner and only slightly on allogeneic PBMCs. This work has demonstrated the feasibility to induce functional Breg-like cells in vitro under stimulation with a bacterial-derived molecule and it opens the way to produce and use Breg-like as a future cellular therapy.

## 2. Results

### 2.1. IL-10-Producing B Cells In Vitro

**Figure 1:**
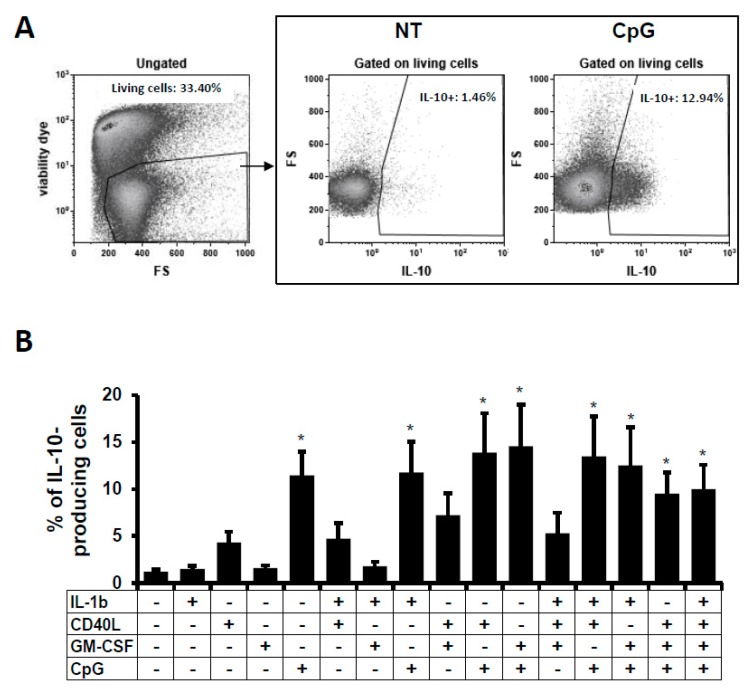
IL-10-producing B cells after B-cell stimulation. B cells were treated for 48 h and analysed by flow cytometry. (**A**) Total IL-10-producing cells were analysed within viable cells using intracellular labelling in non-treated (NT) and CpG-treated B cells (dot plots). Dot plot of one representative experiment out of five is shown. Numbers represent the percentages of living cells and total IL-10-producing B cells. (**B**) Average percentages of IL-10-producing viable non-stimulated or stimulated B cells was analysed. Cultures were performed in the presence of IL-1β, CD40L, GM-CSF, or CpG. Average + SEM of five experiments for each condition are shown. * *p* < 0.05 when comparing non-treated condition versus treated condition.

As commented, IL-10 is the only consensus marker for the majority of functional regulatory B cell subsets. Therefore, we followed the IL-10-producing B cells in this in vitro culture model. Human B cells were isolated from buffy coats and then stimulated for two days with different stimuli such as IL-1β, CD40L, GM-CSF, and CpG alone, or in combination. CD40L and CpG had been described to induce IL-10-producing B cells in vitro [9]. It was also demonstrated that mice lacking IL-1 receptor 1 on B cells have a reduced number of IL-10-producing B cells and develop exacerbated arthritis [15]. Also, low-dose of GM-CSF led to a higher proportion of CD1d^hi^CD5^+^ B cells and B10 cells [18]. IL-10 expression was followed by intracellular labelling in total living B cells (CD20^+^ cells) by flow cytometry (Figure 1A). As can be observed in Figure 1, the IL-10 expression was increased when cells were stimulated with CD40L or CpG in comparison to the non-treated (NT) condition and in comparison to other stimuli used in this experiment. But only the presence of CpG alone or in combination induced a significant increase in IL-10 expression. Combinations of CpG with CD40L or GM-CSF induced the highest frequencies of IL-10-producing B cells (Figure 1B). Summing up, CpG, alone or in combination, was the best stimulus to induce the significant expression of IL-10 in primary human B cell culture.

### 2.2. Phenotype of the B Cells Stimulated In Vitro

**Figure 2:**
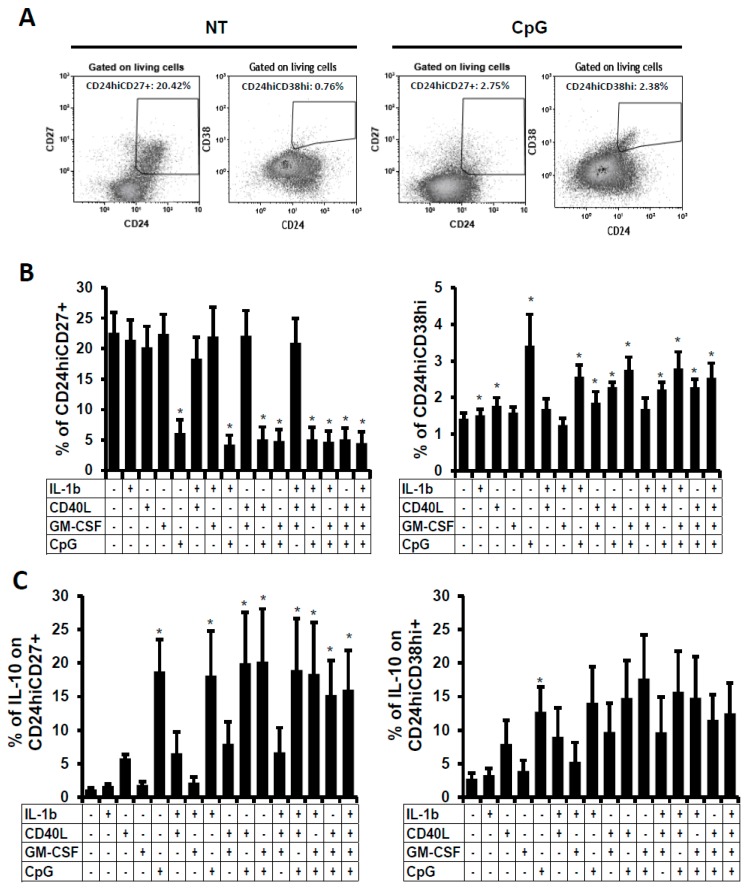
Frequency of two Breg subsets after B-cell stimulation. B cells were non-treated (NT) or treated with IL-1β, CD40L, GM-CSF, or CpG alone, or in combination. 48 h post-stimulation, percentages of (**A**) CD24^hi^CD27^+^ and CD24^hi^CD38^hi^ viable cells were followed. Dot plot of one representative experiment out of five is shown. Numbers represent the percentages of the two Breg subsets. (**B**) Average percentages of CD24^hi^CD27^+^ and CD24^hi^CD38^hi^ non stimulated or stimulated viable B cells. (**C**) Percentages of IL-10 positive cells were detected within CD24^hi^CD27^+^ and CD24^hi^CD38^hi^ viable B cells. Average + SEM of five experiments. * *p* < 0.05 when comparing non-treated condition versus treated condition.

Since some stimuli induced IL-10 expression, which is the hallmark of regulatory cells, we followed the expression of several surface markers that are related to Breg subsets. Thus, frequencies of CD24^hi^CD27^+^ and CD24^hi^CD38^hi^ Breg subsets, which were gated in living cells, were analysed in the B cell culture after two days of stimulation (Figure 2A). We observed that CD27 expression was clearly decreased when cells were stimulated with CpG or with a combination of stimuli containing CpG. As a consequence, the frequency of CD24^hi^CD27^+^ was significantly diminished (Figure 2B, left panel). In parallel, a CD38^+^ subset appeared in CpG-stimulated cells and consequently, the CD24^hi^CD38^hi^ Breg subset was significantly increased when B cells were stimulated with IL-1β, CD40L, or CpG alone, or in combination, even if these increases were slight in frequencies (Figure 2B, right panel). Therefore, even though the frequencies of total IL-10-producing cells were increased in our in vitro model, the frequencies of the two most described Breg subsets were oppositely modified by stimuli, challenging our definition of regulatory B cells followed by these surface markers in an in vitro culture model. Hence, we analysed the expression of IL-10 gating on these two Breg subsets (Figure 2C). We observed that despite the decrease of the most prevalent CD24^hi^CD27^+^subset and the slight increase of the CD24^hi^CD38^hi^ subset (Figure 2B), they were still sensitive to stimulation and were able to be activated and significantly produced IL-10. In the CD24^hi^CD27^+^ subset, an average of 18.8 ± 4.7% (±SEM) of B cells stimulated with CpG expressed intracellular IL-10 in comparison to NT cells that presented an average of 1.2 ± 0.2% (% ± SEM). Identical results were obtained in the CD24^hi^CD38^hi^ subset even though the dispersion of the results was higher. Summing up, in vitro stimulation with stimuli which were described to induce Breg phenotypes, cause a decrease, or minimal increase, in frequencies of the already described Breg subsets, even if these cells were able to express IL-10 at high level.

### 2.3. Phenotype Characterisation of the B Cells Stimulated In Vitro

**Figure 3:**
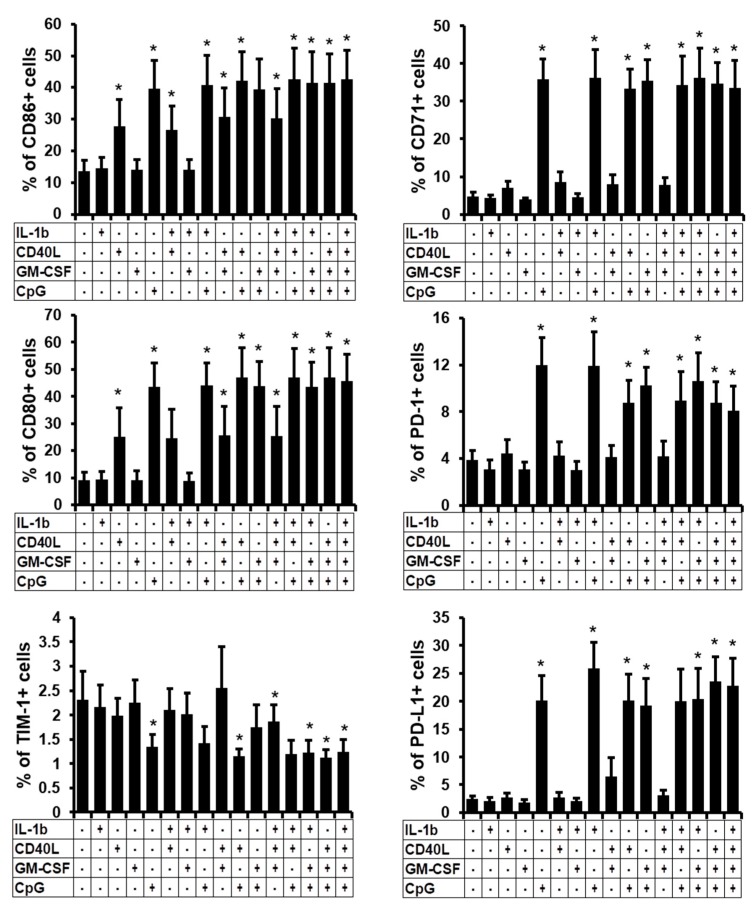
Frequency of Breg-related surface marker subsets. B cells were non-treated (NT) or treated with IL-1β, CD40L, GM-CSF, or CpG alone, or in combination. 48 h post-stimulation, surface markers such as CD86, CD80, TIM-1, CD71, PD-1 and PD-L1 were followed by flow cytometry. Average percentages of these markers are presented. Average + SEM of five experiments. * *p* < 0.05 when comparing non-treated condition versus treated condition.

Because these classical Breg phenotypes under stimulation showed opposite responses, we analysed more deeply the phenotype of the total stimulated B cells. Therefore we followed the expression of CD86, CD80, TIM-1, CD71, PD-1, and PD-L1 on total viable B cells after two days of stimulation (Supplemental Figure S1). CD40L, CpG, and combinations containing these two stimuli induced a significant increase in expression of the Breg functional markers CD86 and CD80 at the surface of total B cells. In parallel, CpG and combinations containing this stimulus, induced significantly the activation marker CD71, the suppressive function marker PD-L1 and the Breg’s modulator PD-1 (Figure 3). However, stimulation did not modify or slightly decreased the expression of TIM-1, which defines TIM1^+^-Breg subset [19], especially when CD40L and CpG were used together. To conclude, apart from the IL-10 expression, stimulation of B cells with CpG alone or in association with other stimuli, induced several markers associated with regulatory function at the surface of the total stimulated B cells.

### 2.4. Suppressive Function of the B Cells Stimulated In Vitro

**Figure 4:**
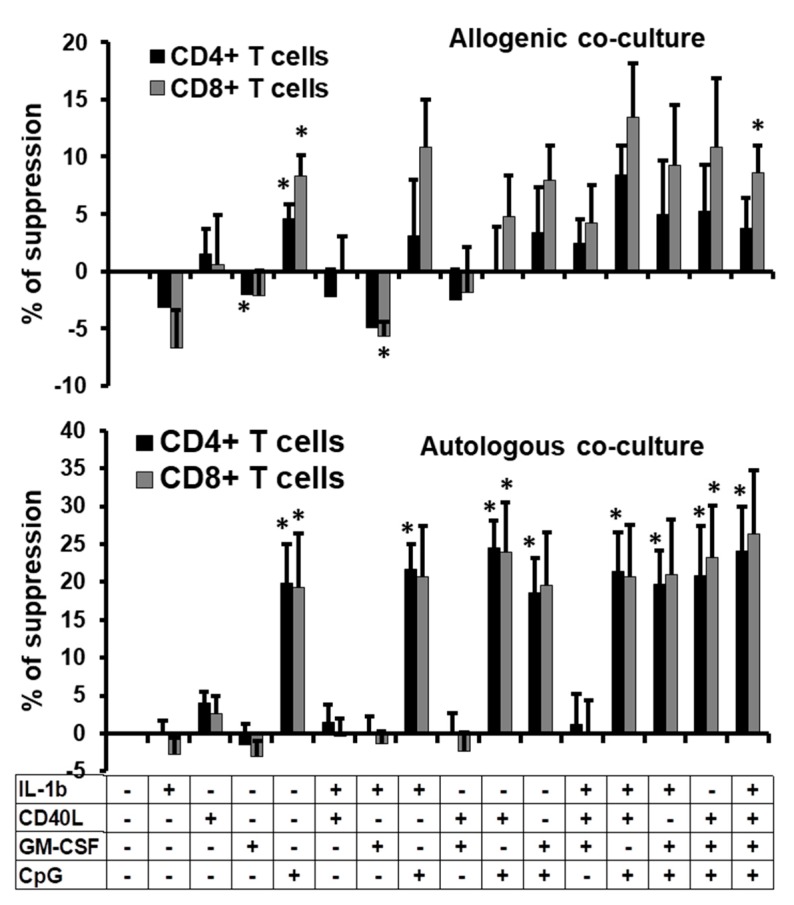
Anti-proliferative function of the stimulated B cells. Percentages of suppression of proliferation of allogenic (upper panel) or autologous PBMCs (lower panel) treated with CFSE and co-cultured with non-treated or treated-B cells (ratio B cells:PBMCs of 2:1) at day 3 post-co-culture. B cells were non-treated or treated with IL-1β, CD40L, GM-CSF, or CpG alone, or in combination for 2 days before co-culture with CFSE+-PBMCs. Average + SEM of the percentages of suppression of CD4^+^ and CD8^+^ T cell proliferation from four different experiments are represented. * *p* < 0.05 when comparing non-treated condition versus treated condition.

Presence of PD-L1 or CD80/CD86 at the surface of B cells and expression of IL-10 intracellularly are hallmarks of regulatory function. Therefore, we tested their possible suppressive ability in a co-culture model. We co-cultured stimulated or non-treated B cells with activated allogeneic carboxyfluorescein succinimidyl ester (CFSE)-labelled PBMCs or autologous CFSE-labelled CD19^-^ PBMCs. We followed the proliferation of CD4^+^ and CD8^+^ T cells from PBMCs by flow cytometry through the loss of CFSE (Supplemental Figure S2). We observed that non-treated B cells were not able to limit the proliferation of CFSE^+^ cells (Supplemental Figure S2). However, when B cells were stimulated with CpG, we observed an apparent diminution in proliferation with a higher peak of non-divided cells (Supplemental Figure S2). Then, we calculated the percentage of suppression exerted by stimulated B cells. We observed that suppression of CFSE^+^ cells was less than 15% when allogeneic PBMCs were used in the co-culture assay, with a higher suppression of PBMC proliferation when cells were co-cultured with B cells stimulated with a mix of IL-1β + CD40L + CpG (Figure 4, upper panel). No significant increase of suppression was observed except when the B cells were treated with CpG or with the mix IL-1β + CD40L + GM-CSF + CpG. Moreover, we observed that in general, CD8^+^ T cells were slightly more sensitive to suppression than CD4^+^ T cells.

However, when autologous CFSE^+^ cells were used in co-culture with CpG-stimulated B cells, we observed about 25% of suppression on the PBMC proliferation and both CD4^+^ and CD8^+^ T cells were equally sensitive to suppression (Figure 4, lower panel). Combinations of stimuli induced the same level of suppression of proliferation. To confirm such results, we also calculated the proliferation index which corresponds to the fold expansion of the cells during co-culture which takes into account the ratio between the final and the starting cellular count [20]. We observed a significant diminution of the proliferation index only when cells were co-cultured in autologous manner and when B cells were treated with CpG alone or in combination (Supplemental Figure S3). In conclusion, not only CpG induced in vitro regulatory-like B-cell phenotypes, but also this stimulus produced functional inhibitory cells that were able to suppress PBMC proliferation in an autologous way.

**Figure 5:**
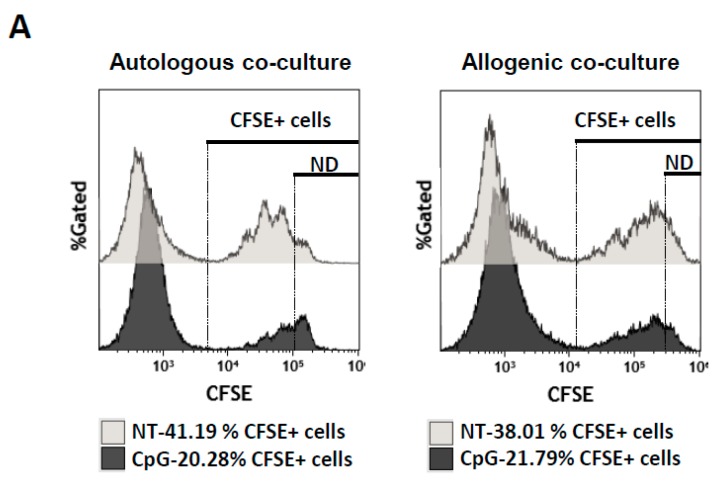

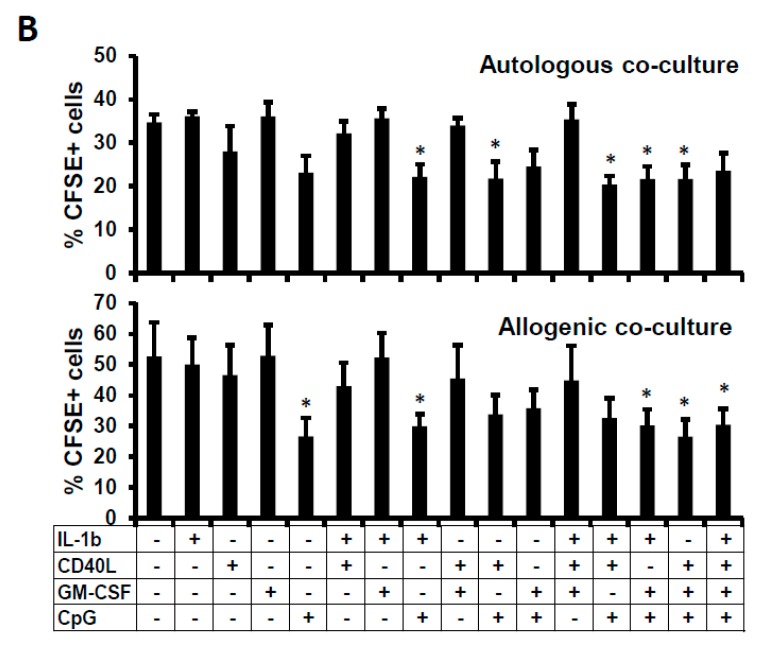
Relation between suppression of proliferation and cell death. (**A**) Histograms of CFSE^+^ cells in the co-culture experiment between non-treated (NT) or CpG-stimulated B cells and CFSE^+^-PBMCs (ratio B cells: CFSE^+^-PBMCs of 2:1) in autologous and allogenic conditions. Numbers represent the percentages of the CFSE^+^ cells in the co-culture. ND for non-divided cells (**B**) Average percentages of CFSE^+^ cells after 3 days of co-culture with B cells either non-treated or treated with IL-1β, CD40L, GM-CSF, or CpG alone, or in combination for 2 days. Average + SEM of four experiments. Loss of CFSE^+^ cells might be due to a lack of cellular proliferation or due to CFSE^+^ cell death. Percentage of suppression of proliferation of CD4^+^ or CD8^+^ T cells was calculated as follows: (frequency of dividing cells = 100 − (proliferation of CFSE^+^-labeled cells in co-culture with stimulated B cells × 100/proliferation of CFSE-labeled T cells in co-culture with non-treated B cells)) [20]. * *p* < 0.05 when comparing non-treated condition versus treated condition.

That diminution of proliferation could be due to two potential mechanisms of suppression related to Breg function: the blockade of proliferation or the induction of cell death. Therefore, we quantified the frequencies of CFSE-labelled PBMCs after three days of co-culture. At day 0, we mixed 50,000 CFSE-labelled PBMCs and 100,000 B cells. Thus 33% of total cells of the co-culture were PBMCs and they were detected by CFSE fluorescence. After three days, we observed that around 30–40% and 40–60% (autologous and allogeneic cells, respectively) of the living cells were CFSE^+^-PBMCs when these cells were co-cultured with NT-B cells (Figure 5A, light grey). These percentages must be related to the ability of PBMCs to proliferate under anti-CD3/anti-CD28 activation and no inhibition was exerted by NT-B cells. However, when PBMCs were co-cultured with CpG-treated B cells, CFSE^+^ cell frequencies were diminished in both autologous and allogeneic co-cultures being significantly diminished when cells were treated with a mix of stimuli containing CpG (Figure 5A, dark grey and Figure 5B). In autologous co-culture, we cannot conclude if the diminution of the PBMC frequency was due to a diminution in proliferation, to PBMC death or both even though the proliferation index showed a clear decrease in fold expansion (Supplemental Figure S3) which could lead us to believe that one of the mechanisms of suppression was related to suppression of proliferation. However, in the allogeneic co-culture, where no evident proliferative suppression was observed in CD4^+^ T cells (Figure 4 and Supplemental Figure S3), we also observed some diminished frequencies of CFSE^+^-PBMCs (Figure 5B). Therefore, we assumed that diminution of PBMC frequencies in co-culture was due to cell death, but cells that had survived were not sensitive to inhibition of proliferation. Summing up, in allogenic culture, we hypothesised that even though treated B cells were not able or only slightly able to diminish the proliferation of PBMCs, they might act through cell death. Finally, in vitro CpG-stimulated B cells were able to limit PBMC expansion in allogeneic or autologous co-cultures through different mechanisms that need to be studied in depth.

## 3. Discussion

Importance of the regulatory function of B cells to control hyper-inflammation is now widely accepted and potential advantages of Breg-based immunotherapy are being investigated. The selection of specific highly functional Breg subsets for cellular therapy might be a challenge and present therapeutic approaches are based on the expansion of immunosuppressive Bregs or generation of Breg in vivo. It is important to point out that almost all the work on Breg generation has been done in mice, which makes work even more difficult on human Bregs since they are less described. Therefore, the major difficulties in the development of Breg-based therapy are due to the unclear human Breg definition and the diversity of Breg-associated suppressive mechanisms [12]. Currently, it is believed that any B-cell subset can acquire regulatory function, thereby different Breg subsets could be induced regarding the microenvironmental factors [15,16]. In this work, we aimed to generate a significant amount of functional human Bregs from peripheral blood. We compared different stimuli (IL-1β, CD40L, GM-CSF, and CpG) in order to identify the factor or the combination of factors that generate functional Bregs in an in vitro model. Surprisingly, although a significant increase in the frequency of IL-10-producing B cells was detected, regulatory-associated phenotype frequencies of these cells showed different changes. Indeed, a diminution of CD24^hi^CD27^+^ and TIM-1^+^-B cell phenotype was observed while the CD24^hi^CD38^hi^ phenotype was marginally increased. We hypothesised that in vitro stimulation could induce some Breg subsets but decrease others highlighting the importance of the choice of the Breg subsets studied. This could be explained by the lack of co-stimulatory T cell contact or by the lack of a proper BCR stimulation since B cells are cultured alone. Moreover, it was shown that antigen-specific recognition by the BCR is important for Breg cell function and development (reviewed in [21]). Therefore, we assumed that because of the lack of physiological condition, in in vitro culture, there might be a selection of some particular Breg subsets. Indeed, stimulated B cells expressed higher percentages of CD80/CD86, CD71 and PD-1, and PD-L1 markers at their surface, all of them related to suppressive function in B cells [6,11,17,22,23,24], but with limited TIM-1, CD27, and CD24 expression.

In the co-culture experiments, and regarding the suppressive ability of these stimulated B cells, it was interesting to note the diminution of the frequency of CFSE^+^-PBMCs when co-cultured with CpG-stimulated B cells, highlighting a possible role of PBMC death. Thus, stimulated B cells could be diminishing the proliferation of effector cells by different mechanisms: suppression of PBMC proliferation and induction of cell death.

In this in vitro model, CpG alone was able to increase a Breg phenotype and function, and no apparent combination of CpG with other stimuli tested in this work induced a higher frequency of Breg-associated function. In vivo, it was demonstrated that IL-1β was able to induce differentiation of IL-10-producing Breg in mice [15]. Contrarily, no IL-10 expression increase was observed after IL-1β stimulation (Figure 1). Otherwise, GM-CSF was described in vivo to expand frequencies and IL-10 production of B cells in an experimental autoimmune mice model of myasthenia gravis [18]. CD40L and CpG have also been described as essential for IL-10-producing B cell generation, [9,25,26]. In our work, CD40L + CpG and GM-CSF + CpG stimuli induced moderately higher frequencies of IL-10-producing B cells. However, these treated cells were not able to suppress T cell proliferation more than the CpG treatment alone. Therefore, CpG seemed to be the major stimulus to induce a Breg-like function in vitro. Further experiments in an in vivo model must be done in the aim to determine if CpG; CD40L + CpG or GM-CSF + CpG-treated cells would be able to conserve their suppressive function in vivo as determined in this in vitro work and also to search the mechanism of the suppression of PBMC proliferation and/or PBMC death.

Generation of autologous Breg-like phenotype in vitro might be the future step for the development of a cellular immune therapy based on Bregs. This work offers the first approach in the generation of human Bregs in vitro and demonstrates that it is feasible to induce functional regulatory B lymphocytes from peripheral human B cells as a possible future cell therapy against autoimmune diseases.

## 4. Materials and Methods

### 4.1. Isolation of B Cells from PBMCs

Peripheral blood mononuclear cells (PBMC) were isolated on a Ficoll-Hypaque density gradient (Rafer, Zaragoza, Spain) from a buffy coat, obtained from the transfusion centre of Madrid following national guidelines. B cells were purified using the CD19-MicroBeads (Miltenyi, Bergisch Gladbach, Germany) and purity was superior to 95%. PBMCs and isolated B cells were cultured with RPMI 1640 medium (Biochrome, Cambourne, UK) supplemented with 5% heat-inactivated FCS, and a mix of antibiotics (125 µg/mL ampicillin, 125 µg/mL cloxacillin and 40 µg/mL gentamicin; Sigma Aldrich, St. Louis, MO, USA).

### 4.2. Culture and Treatment of B Cells

B cells were treated during 48 h before being labelled with surface markers and intracellular IL-10 quantification analysis. Briefly, B cells were treated with CD40L (eBioscience, Waltham, MA, USA; 250 ng/mL), recombinant human IL-1β (ImmunoTools; Friesoythe, Germany; 20 ng/mL), recombinant human GM-CSF (ImmunoTools, 50 ng/mL) or with CpG-B oligodeoxynucleotide-2006 (CpG; Eurogentec SA, Liège, Belgium; 10 µg/mL), alone or in combination. CD40L and CpG were used as positive controls for IL-10-producing B cells, since these stimuli had been described to induce IL-10-producing Breg cells in vitro [9]. NT (non-treated) condition was defined as B cells cultured only with medium as negative control.

### 4.3. Flow Cytometry for Determination of B-Cell Phenotype

Cells were stained to verify the purity of isolated B cells or to define B-cell phenotype by using anti-CD38, anti-CD24, anti-CD27, anti-CD71, anti-CD80, anti-CD86, anti-CD20 (Beckman Coulter, Brea, CA, USA), PD-1 (Miltenyi Biotech), PD-L1 and anti-TIM-1 (eBiosciences) antibodies. For intracellular labelling of IL-10 (Miltenyi Biotech), the B-cell cultures were supplemented with Phorbol 12-myristate 13-acetate (PMA; 10 ng/mL) + Ionomycin (0.25 µg/mL, both from Sigma Aldrich) during 5 h and GolgiStop (BDbiosciences; Franklin Lakes, NJ, USA) during the last 4 h of incubation. Then, cells were washed, surface-stained, stained for viable cells with Fixable Viability Dye eFluor450 (eBiosciences), fixed/permeabilised (Cytofix/Cytoperm, BDbiosciences) and stained for intracellular IL-10. Cells were then analysed by flow cytometry using a Gallios cytometer (Beckman Coulter), and data were analysed using the Kaluza software (Beckman Coulter).

### 4.4. Proliferation Assay

Allogeneic CFSE-labelled PBMCs or autologous CFSE-labelled CD19-depleted PBMCs (50,000 cells) (CFSE from Life technologies; Waltham, MA, USA) were co-cultured with non-treated B cells or stimulated B cells (100,000 cells) and subsequently stimulated with anti-CD3/anti-CD28-coated magnetic beads (Life technologies). After 72 h, cells were stained using anti-CD4 and anti-CD8; the viability of the cells was also followed using 0.5 µg/mL of 7-amino-actinomycin D (7AAD, Sigma-Aldrich). Frequencies of proliferating CD8 and CD4 T cells were determined by flow cytometry following the loss of CFSE signal to determine the suppressive capacity of B cells. To determine the proliferation index, we used the FlowJo-V10 (FlowJo LLC, Ashland, OR, USA) software that allowed us to deconvolute the CFSE profile and calculate the ratio between the final and starting cellular count.

### 4.5. Statistical Analysis

Results are expressed as mean ± SEM. The comparisons between the frequency of IL-10-producing cells, Breg subsets, phenotype of B cells between non-treated and treated B cells were done using the non-parametric Wilcoxon test for paired samples. Statistical comparison of suppressive function and frequency of CFSE^+^ cells was performed by paired Student’s *t*-test. *p* values of < 0.05 were considered statistically significant. All analyses were performed by SPSS 17.0 Inc. (IBM, Armonk, NY, USA).

## Supplementary Materials

The following are available online at http://www.mdpi.com/1422-0067/19/6/1737/s1.
