# Supplementary material for: Generation of Human Breg-Like Phenotype with Regulatory Function In Vitro with Bacteria-Derived Oligodeoxynucleotides
Source: Int J Mol Sci. 2018 Jun 12;19(6):1737. doi: 10.3390/ijms19061737 (PMC6032322; doi:10.3390/ijms19061737)
Supplement: Supplementary file 1 [file ijms-19-01737-s001.pdf]

International Journal of *Molecular Sciences*

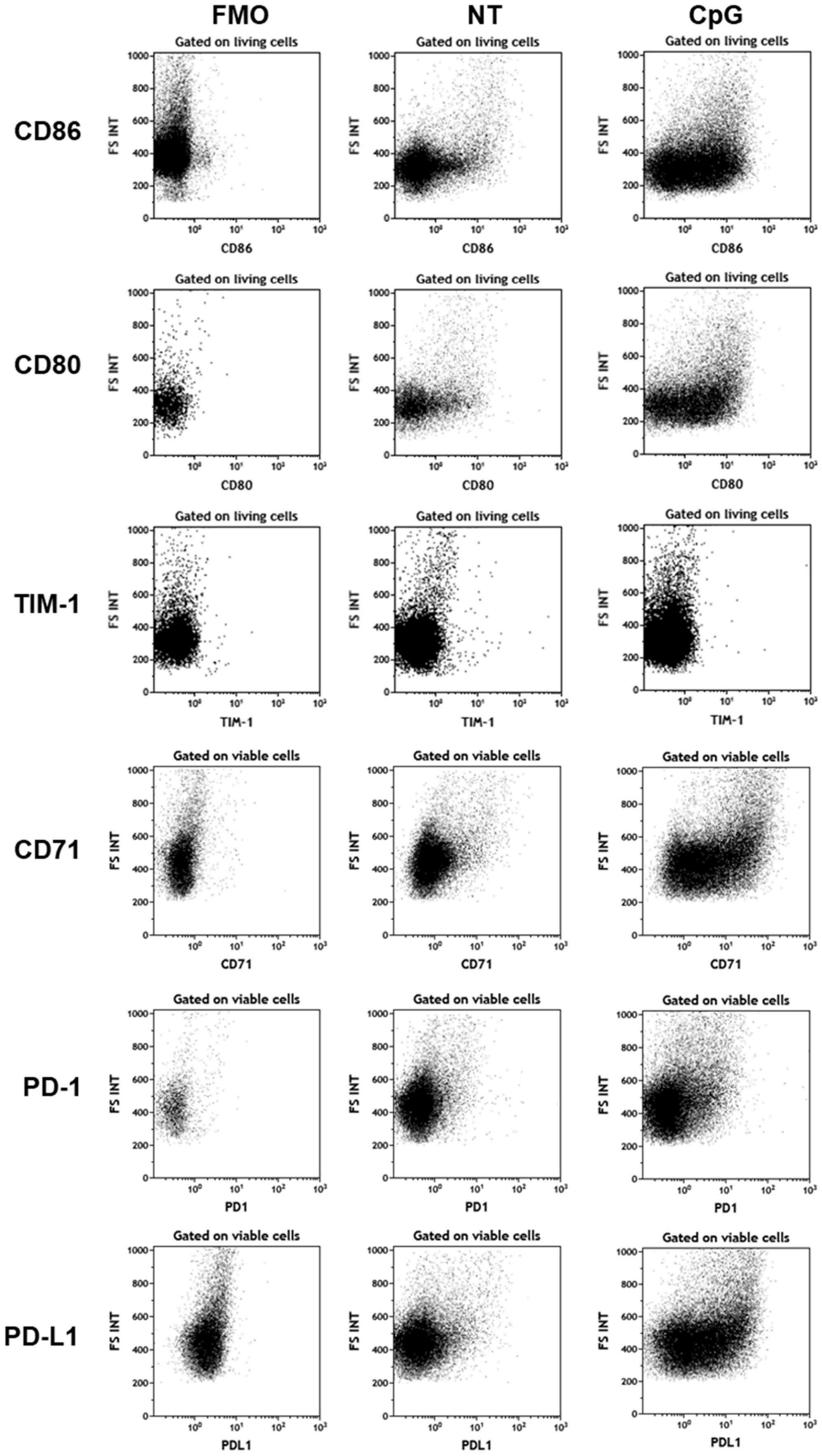

Supplemental Figure S1. Phenotype of total B cells. B cells were treated for 48h and analysed by flow cytometry. CD86, CD80, TIM-1, CD71, PD-1 and PD-L1 expression were analysed within viable cells using surface labelling in non-treated (NT) and CpG-treated B cells (dot plots). Dot plot of one representative experiment out of five is shown. FMO: fluorescence minus one.

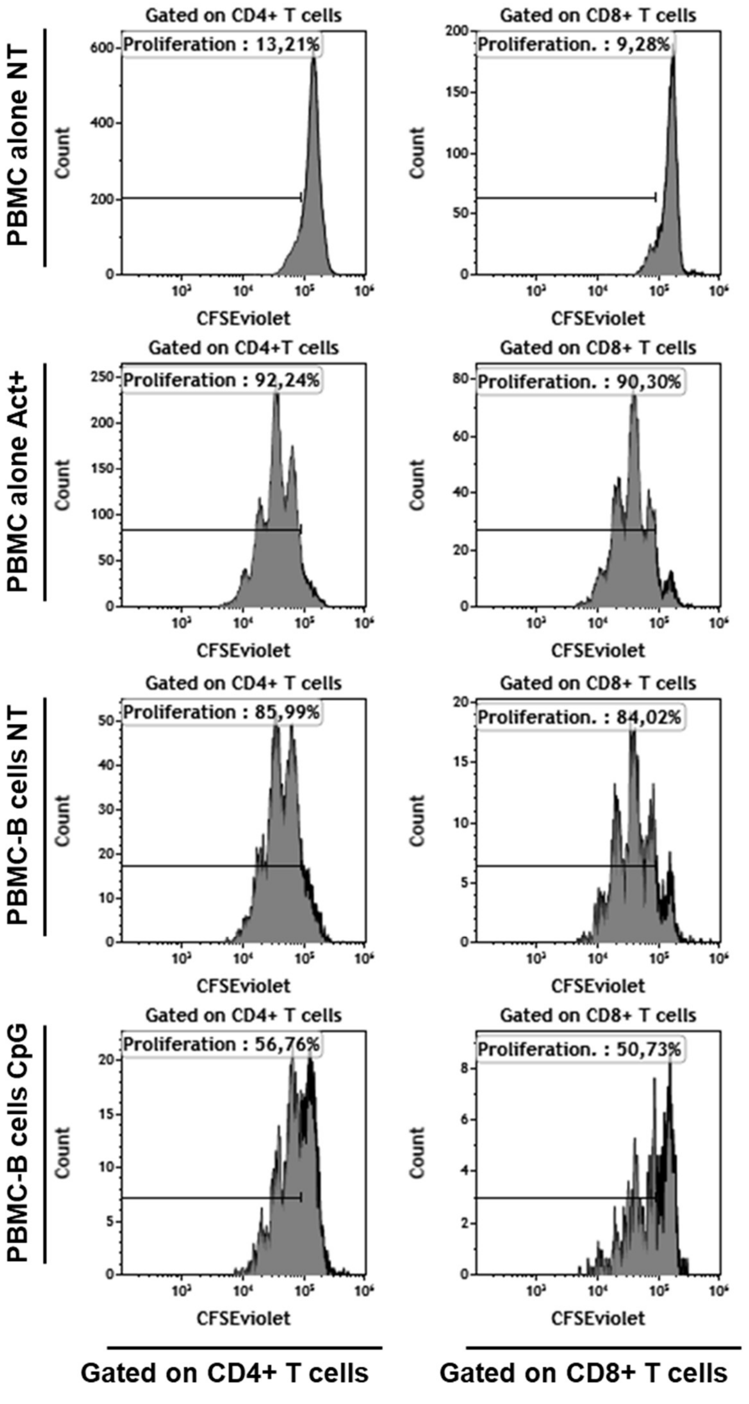

Supplemental Figure S2. Inhibition of the CFSE⁺-PBMC proliferation experiment. Histograms of CD4⁺ and CD8⁺ T-cell proliferation analysed by flow cytometry following the loss of CFSE fluorescence. One representative experiment out of 4 for autologous co-culture experiment is shown. Non-treated PBMCs (NT) alone and activated PBMCs (Act+) alone were used as controls of proliferation. Two co-culture conditions are shown: co-culture of activated PBMCs with NT B cells and co-culture of activated PBMCs with CpG-treated B cells. Numbers represent the percentages of proliferation for CD4⁺ or CD8⁺ T cells.

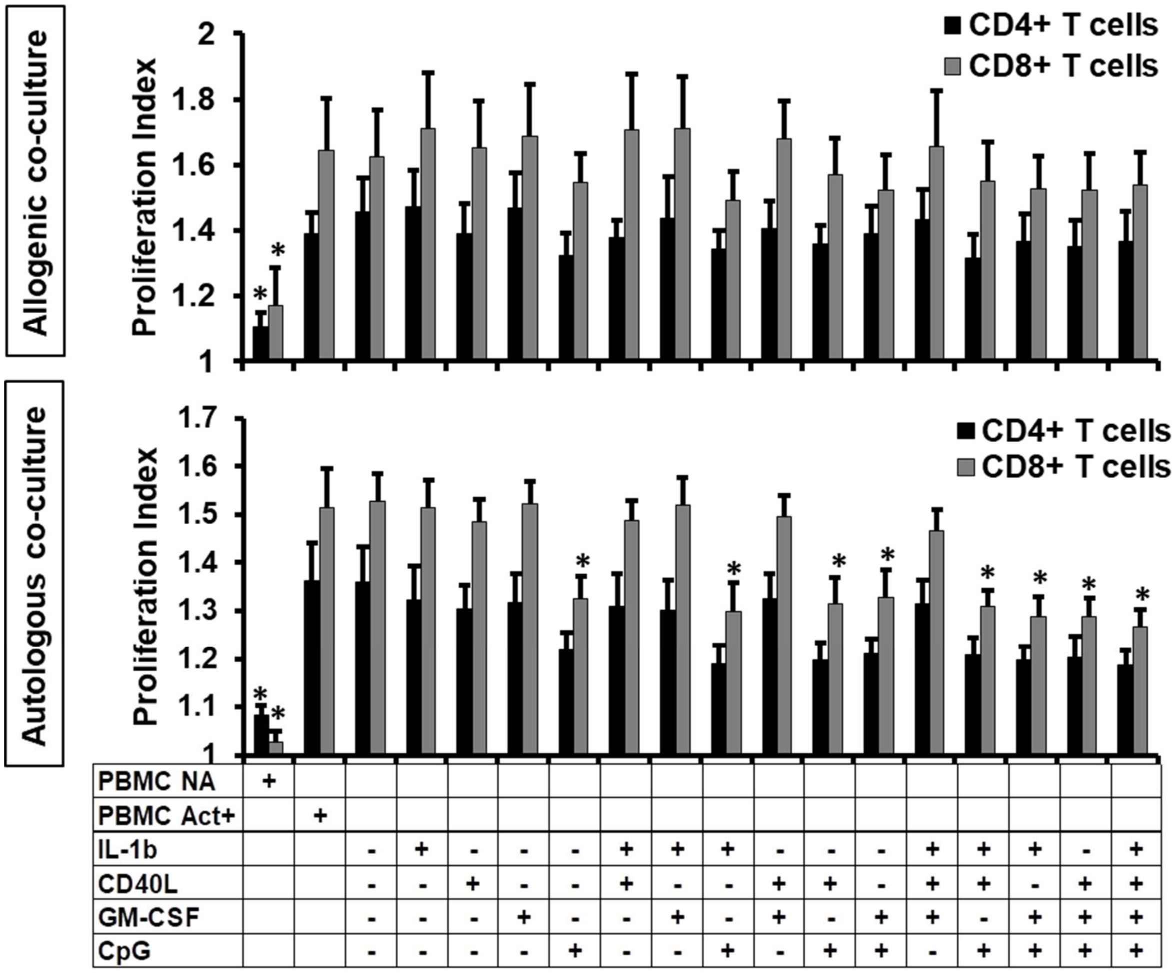

PBMC NA	+																	
PBMC Act+		+																
IL-1b			-	+	-	-	-	+	+	+	-	-	-	+	+	+	-	+
CD40L			-	-	+	-	-	+	-	-	+	+	-	+	+	-	+	+
GM-CSF			-	-	-	+	-	-	+	-	+	-	+	+	-	+	+	+
CpG			-	-	-	-	+	-	-	+	-	+	+	-	+	+	+	+

Supplemental Figure 3. Proliferation index of the B-cell and PBMC co-culture. Histograms of CD4+ and CD8+ T-cell proliferation index analysed by FlowJo-V10 software from flow cytometry data, following the loss of CFSE fluorescence. Proliferation Index corresponds to the fold expansion of the cells during culture (the ratio of final/starting cellular count). Average proliferation index after 3 days of co-culture with B cells either non-treated or treated with IL-1β, CD40L, GM-CSF or CpG alone or in combination for 2 days. Average + SEM of 4 experiments for autologous and allogenic conditions. *=p<0.05 when comparing non-treated condition versus treated condition.
